# Safety and Efficacy of GLP-1 Receptor Agonists in Type 2 Diabetes Mellitus with Advanced and End-Stage Kidney Disease: A Systematic Review and Meta-Analysis

**DOI:** 10.3390/diseases12010014

**Published:** 2024-01-02

**Authors:** Pajaree Krisanapan, Kanokporn Sanpawithayakul, Pattharawin Pattharanitima, Charat Thongprayoon, Jing Miao, Michael A. Mao, Supawadee Suppadungsuk, Supawit Tangpanithandee, Iasmina M. Craici, Wisit Cheungpasitporn

**Affiliations:** 1 Division of Nephrology and Hypertension, Department of Medicine, Mayo Clinic, Rochester, MN 55905, USA; pajareek@tu.ac.th (P.K.); thongprayoon.charat@mayo.edu (C.T.); miao.jing@mayo.edu (J.M.); supawadee.sup@mahidol.ac.th (S.S.); craici.iasmina@mayo.edu (I.M.C.); 2 Division of Nephrology, Department of Internal Medicine, Faculty of Medicine, Thammasat University, Pathum Thani 12120, Thailand; ppatthar@tu.ac.th; 3 Division of Nephrology, Department of Internal Medicine, Thammasat University Hospital, Pathum Thani 12120, Thailand; 4 Division of Endocrinology and Metabolism, Department of Internal Medicine, Faculty of Medicine, Thammasat University, Pathum Thani 12120, Thailand; kanokpor2@tu.ac.th; 5 Department of Clinical Epidemiology, Faculty of Medicine, Thammasat University, Pathum Thani 12120, Thailand; 6 Division of Nephrology and Hypertension, Department of Medicine, Mayo Clinic, Jacksonville, FL 32224, USA; mao.michael@mayo.edu; 7 Chakri Naruebodindra Medical Institute, Faculty of Medicine Ramathibodi Hospital, Mahidol University, Samut Prakan 10540, Thailand; supawit.tan@mahidol.ac.th

**Keywords:** glucagon-like peptide-1 receptor agonist, GLP-1RAs, advanced CKD, CKD stage5, end-stage kidney disease, ESKD, diabetes mellitus, DM, T2DM, liraglutide, dulaglutide, lixisenatide

## Abstract

***Background and Objectives***: Limited evidence exists regarding the safety and efficacy of glucagon-like peptide-1 receptor agonists (GLP-1RAs) in type 2 diabetes mellitus (T2DM) patients with advanced chronic kidney disease (CKD) or end-stage kidney disease (ESKD). Thus, we conducted a systematic review and meta-analysis to assess the safety and efficacy of GLP-1RAs in T2DM patients with advanced CKD and ESKD. ***Materials and Methods***: We performed a systematic literature search in MEDLINE, EMBASE, and Cochrane database until 25 October 2023. Included were clinical trials and cohort studies reporting outcomes of GLP-1RAs in adult patients with T2DM and advanced CKD. Outcome measures encompassed mortality, cardiovascular parameters, blood glucose, and weight. Safety was assessed for adverse events. The differences in effects were expressed as odds ratios with 95% confidence intervals (CIs) for dichotomous outcomes and the weighted mean difference or standardized mean difference (SMD) with 95% confidence intervals for continuous outcomes. The Risk of Bias In Non-randomized Studies—of Interventions (ROBIN-I) tool was used in cohort and non-randomized controlled studies, and the Cochrane Risk of Bias (RoB 2) tool was used in randomized controlled trials (RCTs). The review protocol was registered in the International Prospective Register of Systematic Reviews (CRD 42023398452) and received no external funding. ***Results***: Eight studies (five trials and three cohort studies) consisting of 27,639 patients were included in this meta-analysis. No difference was observed in one-year mortality. However, GLP-1RAs significantly reduced cardiothoracic ratio (SMD of −1.2%; 95% CI −2.0, −0.4) and pro-BNP (SMD −335.9 pmol/L; 95% CI −438.9, −232.8). There was no significant decrease in systolic blood pressure. Moreover, GLP-1RAs significantly reduced mean blood glucose (SMD −1.1 mg/dL; 95% CI −1.8, −0.3) and increased weight loss (SMD −2.2 kg; 95% CI −2.9, −1.5). In terms of safety, GLP-1RAs were associated with a 3.8- and 35.7-time higher risk of nausea and vomiting, respectively, but were not significantly associated with a higher risk of hypoglycemia. ***Conclusions***: Despite the limited number of studies in each analysis, our study provides evidence supporting the safety and efficacy of GLP-1RAs among T2DM patients with advanced CKD and ESKD. While gastrointestinal side effects may occur, GLP-1RAs demonstrate significant improvements in blood glucose control, weight reduction, and potential benefit in cardiovascular outcomes.

## 1. Introduction

Chronic kidney disease (CKD) is a major global public health issue that considerably contributes to the burden of cardiovascular disease (CVD), premature mortality, healthcare expenditure, and decreased quality of life [1,2]. Approximately half of all worldwide incidences of end-stage kidney disease (ESKD) are due to diabetic kidney disease, primarily type 2 diabetes mellitus (T2DM), which is associated with increased CVD and death [3,4,5]. Therefore, the 2022 Kidney Disease Improving Global Outcomes (KDIGO) guidelines emphasized the need for a comprehensive approach to managing people with diabetes and CKD to improve both kidney and cardiovascular outcomes [6]. One of the strategies recommended is the use of glucose-lowering medications with secondary cardiorenal benefits, including sodium-glucose cotransporter-2 inhibitors (SGLT-2is) and glucagon-like peptide-1 receptor agonists (GLP-1RAs), as first- and second-line therapy in T2DM with CKD, respectively [6]. The American Diabetes Association (ADA) also recommends these agents as initial therapy for T2DM patients with CKD, regardless of albuminuria levels [7].

GLP-1 is an incretin hormone, INtestine seCRETion of INsulin secreted by the neuroendocrine L-cells of the intestine after a meal. Natural GLP-1 has a very short plasma half-life (1–2 min) because it is cleaved by the dipeptidyl peptidase-4 (DPP-4) enzyme. Since it is recognized that patients with T2DM have a markedly blunted incretin secretory response, GLP-1RAs were developed to increase the action of GLP-1, thus essentially prolonging its half-life activity [8]. Currently, there are eight GLP-1RAs approved for T2DM treatment, including exenatide (2006), liraglutide (2009), exenatide long-acting release (2011), lixisenatide (2013), albiglutide 2014), dulaglutide (2014), semaglutide (2017), and tirzepatide (2022).

In contrast to sulfonylureas, GLP-1RAs stimulate insulin release in a glucose-dependent pathway and inhibit glucagon release. They also slow gastric emptying time and suppress appetite, thus promoting weight loss [6,9]. GLP-1RAs not only effectively improve glycemic control but have also been shown to reduce major adverse cardiac events (MACE), all-cause mortality, hospitalization due to heart failure, and worsening kidney function among T2DM patients with high cardiovascular risk [6,10,11,12,13,14].

Unlike SGLT-2is, GLP-1RAs may be used in patients with advanced CKD and ESKD, since the elimination of GLP-1RAs depends on both enzymatic pathway (peptidases) and kidney excretion, whereas SGLT-2is are primarily eliminated by the kidneys [15]. Although advanced CKD patients are generally excluded from most randomized controlled trials (RCTs) [10,11,12,13,14,16,17,18,19], there is emerging evidence suggesting that GLP-1RAs may be safe and effective for the treatment of T2DM in advanced CKD/ESKD patients and it may even confer cardiovascular benefits in this high-risk population [20,21,22,23,24,25,26,27]. Thus, we conducted a systematic review and meta-analysis to assess the safety and efficacy of GLP-1RAs in T2DM patients with advanced CKD and ESKD.

## 2. Materials and Methods

### 2.1. Search Strategy and StudyEligilbility

This systematic review protocol was registered with the International Prospective Register of Systematic Reviews (PROSPERO; CRD42023398452). Independently, two investigators (P.K. and K.S.) conducted a systematic search using the Ovid MEDLINE, EMBASE, and Cochrane databases from inception through 25 October 2023. Since the aim was to assess the safety and efficacy of GLP-1RAs in patients with T2DM and advanced CKD and ESKD, the search terms included “GLP-1RAs OR liraglutide OR semaglutide OR dulaglutide OR lixisenatide OR exenatide OR albiglutide OR efpeglenatide” AND “advanced CKD OR ESKD”. The detailed search strategy is provided in the Supplementary Table S1. It is noted that this search included only human studies without language restrictions. Additionally, a manual search of the references in the included studies was conducted to identify any relevant additional studies. The reporting of this systematic review adhered to the standards outlined in the PRISMA Statement [28].

This systematic review included studies if they were observational studies or clinical trials assessing the safety or efficacy outcomes of GLP-1RAs in adults aged 18 years or older with T2DM and advanced CKD, defined as CKD stage 5 (estimated glomerular filtration rate (eGFR) < 15 mL/min/1.73 m^2^) or ESKD undergoing hemodialysis or peritoneal dialysis. The included studies must compare the outcomes of GLP-1RAs to a control group, which were defined as placebo, standard glucose-lowering medications, or lifestyle modification. In the absence of control groups, however, pre- and post-treatment outcomes with GLP-1RAs must be provided. Eligible studies had to report at least one of the following outcomes: all-cause mortality, cardiovascular events (including cardiovascular death, myocardial infarction, stroke, and heart failure), the change in blood pressure or left ventricular hypertrophy, the change in blood glucose, the change in weight or body composition, renal outcomes (the rate of GFR or proteinuria reduction), or any adverse events.

Exclusion criteria were studies that incorporated mixed stages of CKD without conducting a subgroup analysis for advanced CKD or ESKD and studies that reported other treatment outcomes. The studies retrieved underwent independent eligibility reviews conducted by two investigators (P.K. and K.S.). Any discrepancies were resolved through discussions involving all authors.

### 2.2. Data Extraction and Quality Assessment

The following variables were extracted from each included study into the standardized data collection form by each investigator (P.K. and K.S.): study title, names of authors, year of publication, type of study, country of study, number of patients, follow-up duration, age, body mass index (BMI), stage of CKD/ESKD, duration of renal replacement therapy (RRT), duration of T2DM, name and dose of GLP-1RAs, name of control drugs, baseline blood glucose, and outcomes after the treatment with GLP-1RAs or control, including mortality, cardiovascular death, myocardial infarction, stroke, heart failure, blood pressure, left ventricular mass index (LVMI) or cardiothoracic ratio (CTR), pro-B-type natriuretic peptide (pro-BNP), blood glucose, body weight or body composition, the rate of GFR or proteinuria reduction, and any adverse events (such as hypoglycemia, gastrointestinal side effects). The blood glucose fluctuation was defined as the standard deviation of the mean daily blood glucose measured by continuous glucose monitoring (CGM).

Each investigator independently assessed the quality of each study. The Risk of Bias In Non-randomized Studies—of Interventions (ROBIN-I) tool was used in cohort and non-randomized controlled studies [29] (Supplementary Table S2), and the Cochrane Risk of Bias (RoB 2) tool was used in randomized controlled trials (RCTs) (Supplementary Figure S1) [30]. To assess for publication bias, Egger’s test was used in this analysis.

### 2.3. Statisical Analysis

The meta-analyses were conducted using Comprehensive meta-analysis version 3.3.070, developed by Biostat Inc. (Englewood, NJ, USA). The differences in size effects were presented as odds ratios with 95% confidence intervals (CIs) for dichotomous outcomes and the weighted mean difference or standardized mean difference (SMD) with 95% confidence intervals for continuous outcomes. If data was not available in the original articles, we requested it from the investigators or estimated it from the figures. Participants in RCTs were analyzed in intention-to-treat groups.

Heterogeneity was tested using χ^2^ and/or the I^2^ statistic, which was defined as the value of I^2^ above 50% or *p*-value below 0.1. If the test for heterogeneity yielded a significant result, the random-effect model was then applied for meta-analysis. The presence of publication bias was assessed by Egger’s test [31]. Planned subgroup analysis for the primary outcomes stratified by stage of CKD/ESKD was not performed due to insufficient data. In all analyses, statistically significant was defined as a *p*-value <0.05 was considered.

## 3. Results

### 3.1. Study Characteristics

**Figure 1:**
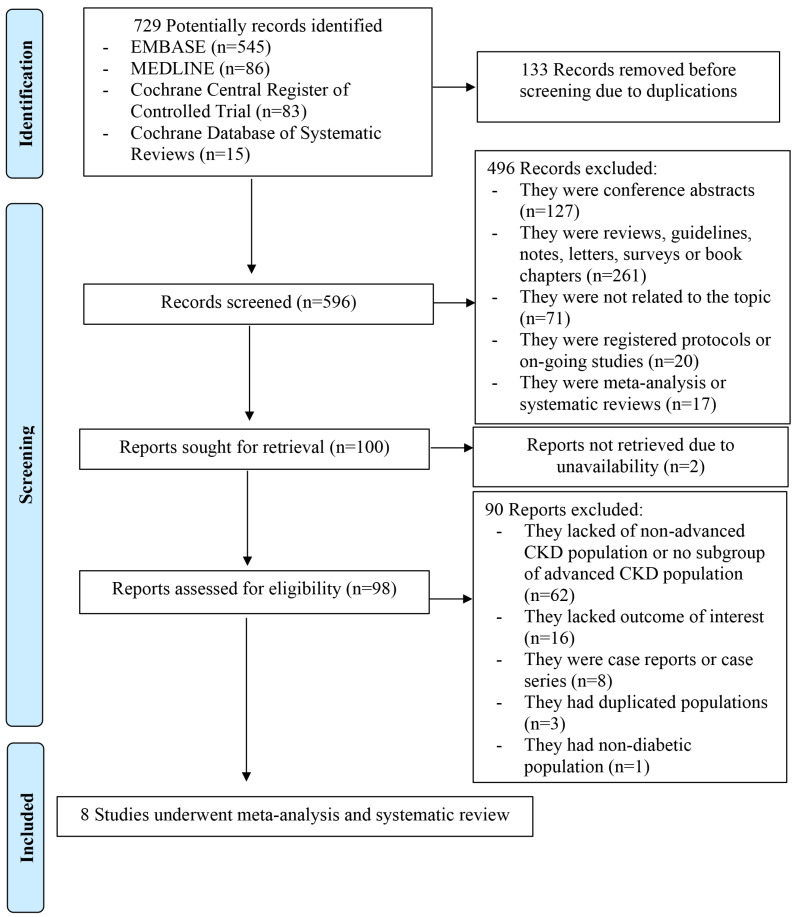
PRISMA flow of search methodology and selection process CCTR, Cochrane Central Register of Controlled Trial; CDSR, Cochrane Database of Systematic Reviews.

A total of 729 potential articles were retrieved by our search strategy (Figure 1), of which 133 were duplicates. The remaining 596 articles were screened for title and abstract, and 496 articles were excluded due to the publication type, lack of topic relevance, and ongoing studies, and 2 articles could not be retrieved due to access unavailability. Therefore, 98 studies underwent full-length review. Of these, 90 studies were excluded due to a lack of an advanced CKD population or a subgroup analysis of the advanced CKD population, the lack of a diabetic population, absent outcomes of interest, case reports or case series, or duplicated population. Ultimately, 8 studies consisting of 27,639 patients were included in this systematic review. These 8 studies were comprised of 4 non-randomized controlled studies [21,23,25,26], 3 retrospective cohorts [32,33,34], and 1 randomized controlled trial (RCT) [22] (Table 1).

Liraglutide was the most frequently used GLP-1RAs across 5 studies, with doses ranging from 0.3 to 1.8 mg daily [21,22,23,33,34]. Dulaglutide was also commonly reported in 3 studies with a fixed weekly dose of 0.75 mg [25,26,33], while lixisenatide was only reported in 1 study without dose description [33]. In 3 studies, GLP-1RAs were prescribed as a monotherapy [21,23,34]. The control group consisted of dipeptidyl peptidase-4 inhibitors (DPP-4is) in 3 studies [23,26,32], insulin in 2 studies [23,25], placebo in 1 study [22], and standard of care (Insulin, oral hypoglycemic drugs, and diet control) in 1 study [21]. Pretreatment data was available in 2 GLP-1RAs studies [33,34]. The median follow-up time was 3 months (IQR 3, 12), ranging from 5 weeks to 4 years.

The majority of patients included in this systematic review were from Asia, with 7 studies conducted in Asia and only 1 from Europe [22]. All 8 studies evaluated ESKD patients undergoing dialysis, while only 2 studies included a subgroup of stage 5 non-dialysis (ND) CKD patients [32,33]. Overall, 54.4% of the patients were male, with a mean age of 64.8 ± 13.0 years and a mean body mass index (BMI) of 24.7 ± 4.6 kg/m^2^. The mean duration of DM and RRT were 9.6 ± 7.4 and 1.4 ± 2.4 years, respectively. Baseline HbA1c was 7.1 ± 1.5%, and glycated albumin was 23.8 ± 7.5%. Notably, 21% of patients had preexisting coronary artery disease in 3 studies [22,32,33].

### 3.2. Efficacy of GLP-1RAs on Mortality

All-cause short-term mortality (1 year) was reported in 6 studies [21,22,23,25,26,34]. Of 105 patients evaluated, no deaths occurred in the GLP-1RAs or control groups within 1 year.

For long-term mortality at 4 years, only 1 cohort study [32] consisting of a total of 27,279 patients reported an all-cause mortality rate of 5.4 (95% CI 4.2–6.6) and 8.0 (95% CI 7.8–8.2) per 100 person-years in the GLP-1RAs group and the control group, respectively, with a hazard ratio (HR) of 0.7 (0.6–0.9, *p*-value = 0.001). However, cardiovascular (CV) mortality rates at 4 years were comparable between GLP-1RAs and the control (2.6 vs. 2.6 per 100 person-years, *p*-value = 0.76 [32].

### 3.3. Efficacy of GLP-1RAs on Cardiovascular Outcomes

**Figure 2:**
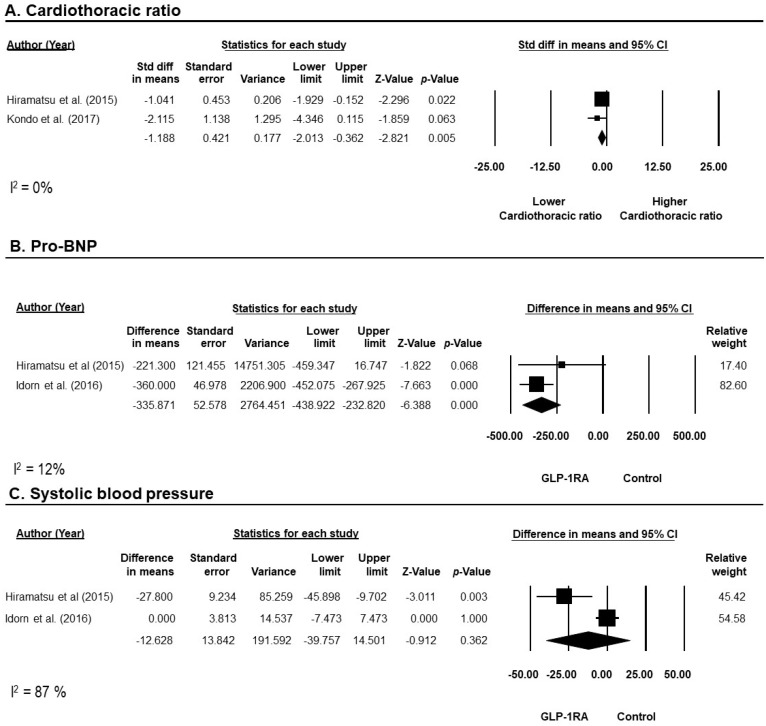
Forrest plot of the efficacy of GLP-1RAs on cardiovascular outcomes represented by cardiothoracic ratio (**A**), Pro-BNP (**B**), and systolic blood pressure (**C**). Studies were identified by name of first author and year of publication. Weighted mean differences were pooled using the random-effects model. CI, confidence interval; GLP-1RAs, glucagon-like peptide 1 receptor agonists; Std diff, standardized difference [21,34,35].

There were no reported cases of myocardial infarction, stroke, or heart failure in any of the included studies. However, short-term (within 1 year) cardiac functions were assessed with left ventricular mass index (LVMI), cardiothoracic ratio (CTR), serum pro-BNP, and systolic blood pressure (SBP). One trial [21] reported that prescribing GLP-1RAs for a year significantly reduced LVMI (−37.7 ± 46.2 g/m^2^) compared to the control, which showed no significant change (−15.0 ± 47.5 g/m^2^). From this meta-analysis, treatment with GLP-1RAs significantly reduced CTR with a standardized mean difference (SMD) of −1.2% (95% CI −2.0, −0.4; I^2^ = 0%; 2 studies [21,34]) as compared to baseline (Figure 2A). Moreover, GLP-1RAs also significantly reduced serum pro-BNP compared to control with an SMD of −335.9 pmol/L (95% CI −438.9, −232.8; I^2^ = 12%; 2 studies [21,22]) (Figure 2B). However, GLP-1RAs did not show a significant decrease in SBP when compared to controls, with notable significant heterogeneity in this meta-analysis (SMD of −12.6 mmHg; 95% CI −39.8, 14.5; I^2^ = 87%; 2 studies [21,22]) (Figure 2C).

### 3.4. Efficacy of GLP-1RAs on Blood Glucose

**Figure 3:**
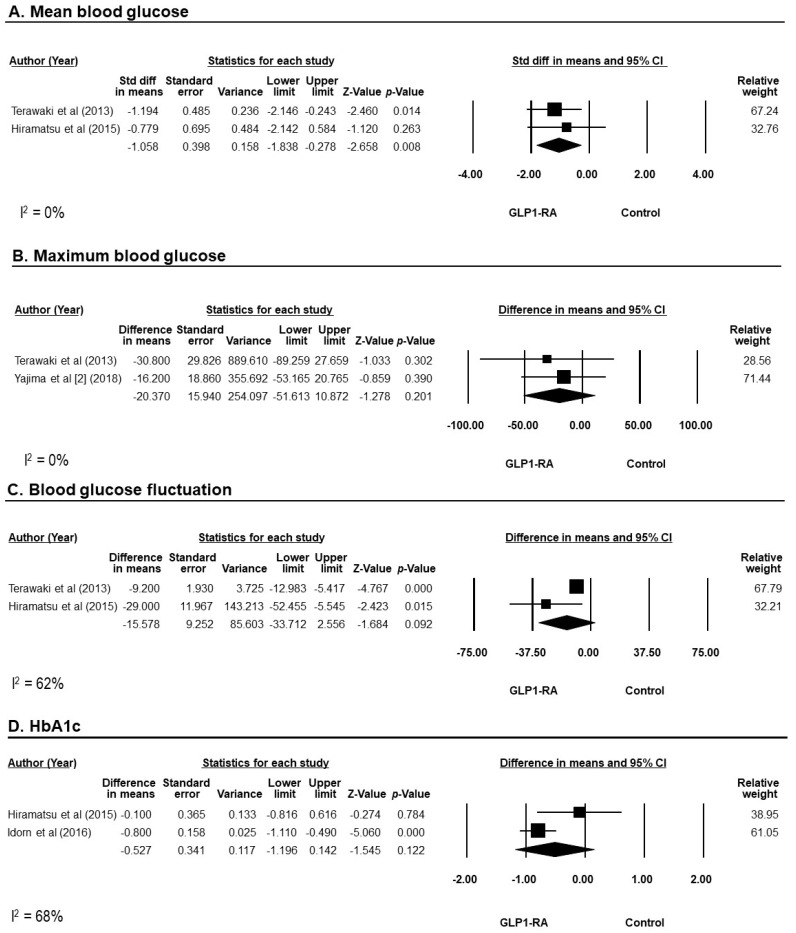
Forrest plot of the efficacy of GLP-1RAs on blood glucose represented by mean blood glucose (**A**), maximum blood glucose (**B**), blood glucose fluctuation (**C**), and HbA1c (**D**). Studies were identified by name of first author and year of publication. Weighted mean differences were pooled using the random-effects model. CI, confidence interval; GLP-1RAs, glucagon-like peptide 1 receptor agonists; Std diff, standardized difference [21,23,26].

For short-term efficacy within 1–3 months, 3 trials assessed GLP-1RAs on blood glucose using CGM [21,23,25]. In this meta-analysis, mean blood glucose was lower in the GLP-1RAs group compared to controls with an SMD of −1.1 mg/dL (95% CI −1.8, −0.3; I^2^ = 0%; 2 trials [21,23]) (Figure 3A). However, GLP-1RAs did not significantly lower maximum blood glucose when compared to controls (SMD of −20.4 mg/dL; 95% CI −51.6, 10.9; I^2^ = 0%; 2 trials [23,25]) (Figure 3B), nor did it decrease blood glucose fluctuation (defined as a mean standard deviation of mean blood glucose, SMD of −15.6 mg/dL; 95% CI −33.7, 2.6; I^2^ = 62%; 2 trials [21,23]) (Figure 3C).

For longer-term efficacy at 3–12 months, HbA1c and glycated albumin were evaluated. GLP-1RAs did not significantly decrease HbA1c when compared to controls, with significant heterogeneity in this meta-analysis (SMD of −0.5 %; 95% CI −1.2, 0.1; I^2^ = 68%; 2 trials [21,22]) (Figure 3D). Only 1 trial reported a change of glycated albumin at 12 months from baseline, which was a decrease of −0.6 ± 1.5% in the GLP-1RAs group and an increase of 0.5 ± 1.8% in the control group [21].

### 3.5. Efficacy of GLP-1RAs on Weight Reduction

Three trials evaluated GLP-1RAs treatment effect on weight at 3–12 months [21,22,26]. From this meta-analysis, GLP-1RAs significantly reduced weight from baseline as compared to controls with an SMD of −2.2 kg (95% CI −2.9, −1.5; I^2^ = 0%) (Supplementary Figure S2).

### 3.6. Efficacy of GLP-1RAs on Renal Outcomes

Only 1 cohort study reported the change of eGFR pre- and post- treatment with GLP-1RAs in advanced-stage CKD (stage 4 and stage 5) [33]. Following treatment with GLP-1RAs, the mean (95% CI) decline in eGFR decreased from −0.6 (−0.6, −0.5) to −0.1 (−0.2, −0.1) mL/min/1.73 m^2^/month.

### 3.7. Safety of GLP-1RAs

**Figure 4:**
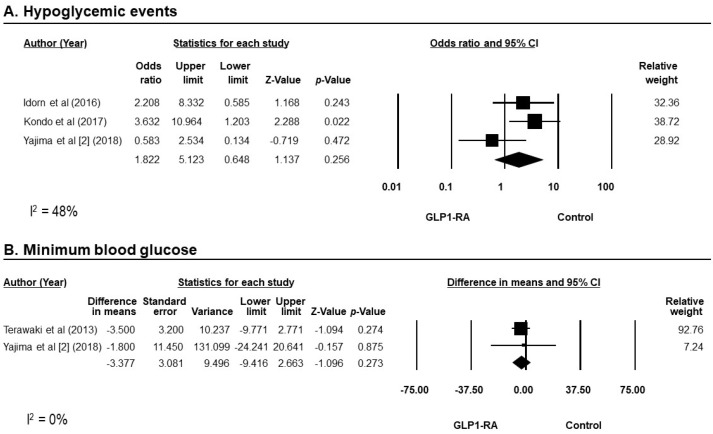
Safety of GLP-1RAs on blood glucose lowering represented by hypoglycemic events (**A**) and minimum blood glucose (**B**). Studies were identified by name of first author and year of publication. Weighted mean differences and odds ratio were pooled using the random-effects model. CI, confidence interval; GLP-1RAs, glucagon-like peptide 1 receptor agonists [23,26,34,35].

The safety of GLP-1RAs on blood glucose lowering was assessed via hypoglycemia events and minimum blood glucose. In this meta-analysis, GLP-1RAs did not significantly increase the risk of hypoglycemic events in advanced CKD patients as compared to controls, with a pooled odds ratio of 1.8 (95% CI 5.1, 0.6; I^2^ = 48%; 3 trials [22,25,34]) (Figure 4A). Likewise, GLP-1RAs did not significantly lower minimum blood glucose more than controls (SMD of −3.4 mg/dL; 95% CI −9.4, 2.7; I^2^ = 0%; 2 trials [23,25]) based on CGM measurements (Figure 4B).

Of 135 patients evaluated in 6 studies [21,22,23,25,26,34], 6 (8.6%) serious adverse events (SAEs) were reported in the GLP-1RAs group versus only 1 event (1.5%) in the control group. Chest pain and infection clotted arteriovenous (AV) fistula, catheter infection, lumbar spinal stenosis, and acute appendicitis were the SAEs reported with GLP-1RAs primarily in 1 RCT [22].

Gastrointestinal (GI) side effects were reported in 2 trials with a total of 54 participants evaluated [21,22]. Nausea was reported with an incidence rate of 30.1 days/1000 patient-day in GLP-1RAs and 7.9 days/1000 patient-day in the control. The incidence rates of vomiting were 10.7 days/1000 patient-day in GLP-1RAs and 0.3 days/1000 patient-day in the control. Of 24 patients, the incidence rates of reduced appetite were 183 days and 10.7 days per 1000 patient-day in GLP-1RAs and the control, respectively [22].

Injection site reactions were primarily reported in only 1 RCT, which occurred in 14.3% of the GLP-1RAs group and 40% of the control group [22].

## 4. Evaluation of Publication Bias

A funnel plot was not constructed due to the paucity of studies included. Conventionally, it is recommended that tests for asymmetry in funnel plots be employed only when the number of study groups equals or exceeds 10. Owing to this limitation in the number of studies, the statistical power of the test remains inadequate to effectively discern between random variation and actual asymmetry [36]. Egger’s regression asymmetry test showed no publication bias with *p* > 0.05 for all analyses.

## 5. Discussion

A recent meta-analysis by Sattar et al. [14] demonstrated that GLP-1RAs reduce the composite MACE, which consists of cardiovascular death, myocardial infarction, and stroke, by 14% in individuals with T2DM. In addition, GLP-1RAs have been shown to decrease all-cause mortality, hospitalization for heart failure, and composite kidney outcomes without any increased risk of adverse events [14]. However, it is highlighted that the RCTs included in this aforementioned meta-analysis had a small proportion of patients (14.6%) with significant CKD (eGFR < 60 mL/min/1.73 m^2^), and none of them had advanced CKD (eGFR < 15 mL/min/1.73 m^2^) or ESKD [10,11,12,13,16,17,18,19,37,38]. Since kidneys are responsible for the partial excretion of GLP-1RAs, it is not surprising that these advanced CKD patients were initially excluded from RCTs [15].

While liraglutide and dulaglutide are the most commonly used GLP-1RAs among patients with advanced CKD and ESKD in our review, there have been some case reports that have demonstrated the favorable effectiveness and safety of semaglutide in individuals with ESKD [20,24,39]. In contrast to short-acting GLP-1RAs and exenatide, the majority of liraglutide, dulaglutide, and semaglutide is primarily eliminated through the peptidases enzymatic pathway, with only a small percentage of the drug excreted in the kidneys [15]. Therefore, the findings of our meta-analysis showing that the use of GLP-1RAs in patients with advanced CKD did not significantly increase the risk of hypoglycemia appears rational.

Despite a higher incidence of SAEs in the GLP-1RAs group compared to the control (8.6% vs. 1.5%), it is noted that all reported SAEs cases were primarily from only one RCT [22], and no such cases were reported in the other studies included in this systematic review. Furthermore, the authors of that RCT stated that none of the SAEs were related to the GLP-1RAs and that all patients fully recovered without permanent injury [22]. GI side effects, however, were reported more frequently in ESKD patients treated with GLP-1RAs, with a 3.8-fold increased risk of nausea and a 35.6-fold increased risk of vomiting when compared to the control group. A previous meta-analysis of GLP-1RAs in a general population without significant CKD similarly found that liraglutide increased the risk of nausea and vomiting, with an odds ratio (OR) range between 3.6–5.3 and 6.1–6.9, respectively [40]. Thus, although the use of GLP-1RAs in patients with advanced CKD and ESKD may carry a higher risk of vomiting compared to a general population, there were also no reported cases of severe dehydration or other sequelae, even in those with the most severe cases [22].

The benefit of GLP-1RAs on long-term mortality outcomes was assessed primarily in one nationwide cohort study [32]. Compared to the control group, GLP-1RAs decreased all-cause mortality in advanced CKD patients by 30%, significantly higher than the 12% associated in patients without significant CKD [14]. It is noted that the greatest reduction in all-cause mortality among advanced CKD and ESKD patients was predominantly driven by sepsis- and infection-related deaths rather than CV deaths, which are commonly observed in those T2DM patients in general [14,32]. Considering that diabetes and CKD are both recognized as diseases that increase susceptibility to infection and are associated with poor outcomes following sepsis [32,41,42], it is plausible that incretin-based therapy could improve outcomes by decreasing excessive inflammation and microvascular thrombosis in sepsis via GLP-1 receptor activation [32,43,44]. Conversely, despite being classified as an incretin-based therapy, DPP-4is have been associated with higher infection-related mortality [32]. This association with increased mortality in diabetic patients may have resulted from COVID-19 infection during the pandemic [45]. To determine the long-term benefit of GLP-1RAs on CV mortality, more studies with longer follow-up times are needed.

The efficacy of cardiovascular outcomes was evaluated using cardiac function tests rather than standard MACE due to the limited events during the follow-up duration of the studies included. In this meta-analysis, GLP-1RAs significantly reduced CTR from baseline, pro-BNP levels and LMVI in ESKD patients compared to the control group. Notably, these beneficial effects of GLP-1RAs on left ventricular (LV) function cannot be solely attributed to changes in blood pressure, as shown by a nonsignificant change in SBP in this meta-analysis, but rather due to both glucose-dependent and non-glucose-dependent mediated pathways [46]. A recent study by Nikolaidis et al. also demonstrated an improvement in LV function in patients with acute MI and severe systolic dysfunction after successful primary angioplasty within 72 h of GLP-1RAs infusion [46]. Altogether, the available evidence may support the hypothesis that GLP-1RAs improve CV outcomes via numerous favorable cardiac effects, including increased glucose uptake, decreased metabolism of free fatty acid and accumulation of triglyceride, improved production of nitric oxide (NO), enhanced reduction of vasorelaxation/afterload, and increased cardiac contractility [15,47,48].

The effect on blood glucose was evaluated in this meta-analysis, and it was surprising to find that GLP-1RAs treatment did not significantly lower HbA1c compared to the control group. One possible explanation for this lack of significance is that the mean baseline HbA1c level of the included patients was 6.2 ± 0.7%, which falls within the acceptable range for most individuals with diabetes [6,49]. Given that GLP-1RAs stimulate insulin release in a glucose-dependent manner, its effectiveness may not be apparent in populations with well-controlled blood glucose [9,15,50]. Another possible reason for the lack of statistical difference in HbA1c is that the differences in blood glucose between GLP1-RAs vs. controls, while statistically significant, are very subtle with an SMD of −1.1 mg/dL and in the setting of concurrent alternative hyperglycemic drug treatment depending on study control group. Hence, this between-group difference is too small to affect the HbA1c. Finally, HbA1c measurements may have limited accuracy in patients with advanced CKD due to various factors such as anemia, treatment with erythropoiesis-stimulating agents (ESAs), or iron supplements, which can lead to underestimation of HbA1c levels [6]. Although GLP-1RAs did not lower the maximum blood glucose level in our meta-analysis, it is noteworthy that the treatment did lead to a significant improvement in postprandial glucose excursion in one study [21].

Continuous glucose monitoring (CGM) is another glucose measurement tool recommended by KDIGO in diabetic patients with advanced CKD and ESKD. This meta-analysis revealed that GLP-1RAs significantly reduced mean blood glucose levels compared to the control group. Despite each individual study showing a significant improvement in glucose fluctuation with GLP-1RAs, GLP-1RAs did not decrease blood glucose variability. This may be due to the limited numbers of patients and high heterogeneity among the included studies, making it difficult to detect the glucose fluctuations [21,23].

As compared to a previous meta-analysis, weight reduction with GLP-1RAs resulted in less benefit for patients with advanced CKD than those in the general population, with an SMD of −2.2 kg (95% CI −2.9, −1.5) and −7.1 kg (95% CI −9.2, −5.0), respectively [51]. This discrepancy can be attributed to the greater BMI of the participants in the previous study than ours. It is also possible that patients with advanced CKD and uremia have multiple inflammatory milieu and comorbidities that result in increased fatigue, decreased activity, and reduced metabolism.

We found limited evidence regarding the impact of GLP-1RAs on renal outcomes, as only one cohort study reported a significant reduction in eGFR decline in stage 5 CKD patients. An ongoing FLOW trial is currently underway to evaluate the effects of semaglutide on kidney outcomes in individuals diagnosed with T2DM and CKD, with anticipated results slated for release in 2024. It is pertinent to note, however, that the preliminary mean eGFR in this study stood at 47 mL/min/1.73 m^2^, an indicative of a non-advanced CKD or ESKD population [35]. Therefore, further studies are needed to draw a definitive conclusion on the benefits of GLP-1RAs on renal outcomes in this patient population.

There are several limitations in our systematic review and meta-analysis that should be acknowledged. Firstly, the inclusion of a few studies with a limited number of patients in each analysis resulted in significant heterogeneity among the included studies on certain outcomes of interest, including SBP, HbA1c, and hypoglycemia. Secondly, the short-term follow-up period of the included studies, most of which were within one year, limits our ability to assess long-term outcomes such as mortality and MACE. Thirdly, the included studies predominantly involved Asian populations, which may limit the generalizability of our findings to other ethnic groups. This study did not include patients with ESKD who were transplanted, nor did it have sufficient numbers to perform a subgroup analysis on the type of dialysis modality and the associated impact of GLP-1RAs. Finally, only a few studies evaluated blood glucose levels with CGM, and none of them reported self-monitoring blood glucose (SMBG), which is more reliable than HbA1c in patients with advanced CKD and ESKD [6]. Thus, future studies with larger sample sizes, longer follow-up periods, and more diverse populations are needed to confirm our findings and address these limitations. Despite these limitations, our systematic review provides important insights into the safety and efficacy of GLP-1RAs among T2DM patients with advanced CKD.

To the best of our knowledge, this is the first systematic review and meta-analysis that evaluates the safety and efficacy of GLP-1RAs among T2DM patients with advanced CKD. Our finding demonstrated that the use of GLP-1RAs in patients with advanced CKD and ESKD is generally safe, except for an increased risk of vomiting that necessitates close monitoring following drug initiation. Furthermore, these patients exhibit favorable cardiovascular outcomes, blood glucose control, and weight reduction from GLP-1RAs, similar to the benefits observed in T2DM patients without significant CKD. Nonetheless, further studies with long-term follow-up are needed to confirm the benefit of GLP-1RAs on mortality and MACE.

## 6. Conclusions

Our study summarizes the safety and efficacy size of GLP-1RAs among T2DM patients with advanced CKD and ESKD. While GLP-1RAs might increase the risk of GI side effects, GLP-1RAs demonstrate significant improvements in blood glucose control, weight reduction, and potential benefit in cardiovascular outcomes.

## Figures and Tables

**Table 1:** Characteristics of included studies.

Author (Year)	Terawaki et al. [23] (2013)	Hiramatsu et al. [21] (2015)	Idorn et al. [22] (2016)	Kondo et al. [34] (2017)	Yajima et al. [1] (2018)	Yajima et al. [2] (2018)	Hirose et al. [33] (2021)	Chen et al. [32] (2022)
**Study type**	Crossover controlled trial	Non-randomized controlled study	Randomized controlled trial	Retrospective cohort	Non-randomized controlled study	Non-randomized controlled study	Retrospective cohort	Retrospective cohort
**Site**	Japan, single center	Japan, single center	Denmark, multicenter	Japan, single center	Japan, single center	Japan, single center	Japan, national database	Taiwan, national database
**GLP-1RA name**	Liraglutide	Liraglutide	Liraglutide	Liraglutide	Dulaglutide	Dulaglutide	61.5% Dulaglutide, 36.5% Liraglutide, 2% Lixisenatide	N/A
**GLP-1RA dosage**	0.3 mg daily	0.6–0.9 mg daily	Titrate to a maximum dose of 1.8 mg daily	0.3–0.9 mg daily	0.75 mg weekly	0.75 mg weekly	N/A	N/A
**Use of GLP-1RA**	Single therapy	Single therapy	Add on therapy	Single therapy	Add on therapy	Add on therapy	Both single and add on therapy	Both single and add on therapy
**Concomitant insulin used with GLP-1RA, %**	0	0	80	0	100	100	34.1	16.3
**Control**	Vildaglipin, alogliptin, and insulin	Standard therapy	Placebo	None	Teneligliptin	Insulin	None	DPP-4i
**Total N** **GLP1-RA** **Control**	10 10 10	30 15 15	24 ^a^ (20 ^b^) 14 ^a^ (10 ^b^) 10	5 5 0	21 11 10	15 15 15	255 255 0	27,279 701 26,578
**Male, n (%)**	7 (70.0)	22 (73.3)	17 (85.0)	4 (80.0)	16 (76.2)	13 (86.7)	167 (65.5)	14,789 (54.2)
**Age, year**	62.9B ± 4.3	67.6 ± 7.0	67.1 ± 3.8	67.8 ± 4.3	68 (61, 72) ^c^	72 (66, 79) ^c^	66.5 ± 11.6	64.8 ± 13.0
**Body mass index, kg/m^2^**	23.0 ± 1.5	24.8 ± 3.9	31.6 ± 2.4	23.2 ±1.2	23.1 (21.6, 26.3) ^c^	23.6 (22.9, 25.3) ^c^	24.5 ± 5.1	N/A
**Stage of advanced CKD**	ESKD undergoing HD	ESKD undergoing PD	ESKD	ESKD undergoing HD	ESKD undergoing HD	ESKD undergoing HD	Stage 5 ND and ESKD	Stage 5 ND and ESKD
**Duration of RRT**	4.1 ± 1.1 years	10 ± 9.3 months	N/A	N/A	13.5 (3.7, 30.8) months ^c^	12 (2, 82) months ^c^	N/A	N/A
**Duration of DM, years**	25.4 ± 2.3	17.6 ± 12	14.2 ± 2.4	N/A	N/A	22 (18, 32) ^c^	6.9 ± 6.9	N/A
**Baseline HbA1c, %**	N/A	5.9 ± 0.8	6.7 ± 0.4	6.0 ± 1.0	N/A	6.2 (5.3, 6.8) ^c^	7.4 ± 1.6	N/A
**Baseline glycated albumin, %**	24.1 ± 1.5	16.9 ± 0.4	N/A	N/A	22.3 (17.6, 25.1) ^c^	21.8 (17.9, 25.1) ^c^	24.9 ± 8.3	N/A
**Baseline Hemoglobin, mg/dL**	N/A	N/A	N/A	10.1± 0.5	10.4 (9.3, 11.6) ^c^	10.6 (9.5, 12.5) ^c^	N/A	N/A
**Comorbidities** **CAD, n (%)** **Stroke, n (%)** **HF, n (%)** **DR, n (%)**	N/A N/A N/A 0 (0)	N/A	7 (35) N/A N/A 9 (45)	N/A	N/A	N/A	87 (34.1) 29 (11.4) 124 (48.6) 51 (20)	5722 (21.0) 3387 (12.4) 4751 (17.4) N/A
**Follow-up time**	3 months	12 months	3 months	3 months	6 months	5 weeks	N/A	4 years ^d^
**Source of funding**	Ministry of health, labor and welfare, Japan	None	Drug company (Novo Nordisk)	None	N/A	N/A	Drug company (Eli Lilly)	Chang Gung memorial hospital and ministry of science and technology, Taiwan

^a^ intention to treat analysis, ^b^ per protocol analysis, ^c^ median (IQR 25, 75), ^d^ only for survival analysis. CAD, coronary artery disease; CKD, chronic kidney disease; DPP-4i, dipeptidyl peptidase IV inhibitors; DR, diabetic retinopathy; ESKD, end-stage kidney disease; HD, hemodialysis; HF, heart failure; ND, non-dialysis; N, number of populations; N/A, no data available; PD, peritoneal dialysis; RRT, renal replacement therapy.

## Data Availability

The data supporting this study can be found in the original publication, reports, and preprints referenced in the citations.

## Supplementary Materials

The following supporting information can be downloaded at: https://www.mdpi.com/article/10.3390/diseases12010014/s1, Figure S1: Cochrane Risk of Bias (RoB 2) Tool Utilization in Randomized Controlled Trials (RCTs). Figure S2: Weight Reduction Efficacy of GLP-1RAs. This figure presents a meta-analysis of weight change outcomes in patients treated with GLP-1RAs, compared to controls. Table S1: Data Extraction Form Used for Study Inclusion. Table S2: Risk of Bias In Non-randomized Studies—of Interventions (ROBIN-I) Assessment Results.
