# Supplementary material for: Safety and Efficacy of GLP-1 Receptor Agonists in Type 2 Diabetes Mellitus with Advanced and End-Stage Kidney Disease: A Systematic Review and Meta-Analysis
Source: Diseases. 2024 Jan 2;12(1):14. doi: 10.3390/diseases12010014 (PMC10814593; doi:10.3390/diseases12010014)
Supplement: Supplementary file 1 [file diseases-12-00014-s001.zip › diseases-2750929-supplementary.pdf]

PRISMA 2020 for Abstracts Checklist

Section and Topic	Item #	Checklist item	Location where item is reported
**TITLE**			
Title	1	Identify the report as a systematic review.	Title (page 1, line 1-4)
**ABSTRACT**			
Abstract	2	See the PRISMA 2020 for Abstracts checklist.	Please see PRISMA 2020 abstract checklist
**INTRODUCTION**			
Rationale	3	Describe the rationale for the review in the context of existing knowledge.	Introduction (page 2, line 54-92)
Objectives	4	Provide an explicit statement of the objective(s) or question(s) the review addresses.	Introduction (page 2, line 84-92)
**METHODS**			
Eligibility criteria	5	Specify the inclusion and exclusion criteria for the review and how studies were grouped for the syntheses.	Search strategy and study eligibility (page 3, line 107-124)
Information sources	6	Specify all databases, registers, websites, organisations, reference lists and other sources searched or consulted to identify studies. Specify the date when each source was last searched or consulted.	Search strategy and study eligibility (page 2-3, line 95-106)
Search strategy	7	Present the full search strategies for all databases, registers and websites, including any filters and limits used.	Search strategy and study eligibility (page 2-3, line 95-106), Table s1 in supplement
Selection process	8	Specify the methods used to decide whether a study met the inclusion criteria of the review, including how many reviewers screened each record and each report retrieved, whether they worked independently, and if applicable, details of automation tools used in the process.	Search strategy and study eligibility (page 3, line 107-124), Data extraction and quality assessment (page 3, line 126-142)
Data collection process	9	Specify the methods used to collect data from reports, including how many reviewers collected data from each report, whether they worked independently, any processes for obtaining or confirming data from study investigators, and if applicable, details of automation tools used in the process.	Data extraction and quality assessment (page 3, line 126-142)
Data items	10a	List and define all outcomes for which data were sought. Specify whether all results that were compatible with each outcome domain in each study were sought (e.g. for all measures, time points, analyses), and if not, the methods used to decide which results to collect.	Search strategy and study eligibility (page 2-3, line 107-124)
	10b	List and define all other variables for which data were sought (e.g. participant and intervention characteristics, funding sources). Describe any assumptions made about any missing or unclear information.	Data extraction and quality assessment (page 3, line 126-142)
Study risk of bias assessment	11	Specify the methods used to assess risk of bias in the included studies, including details of the tool(s) used, how many reviewers assessed each study and whether they worked independently, and if applicable, details of automation tools used in the process.	Statistical analysis (page 4, line 153-154)

Section and Topic	Item #	Checklist item	Location where item is reported
Effect measures	12	Specify for each outcome the effect measure(s) (e.g. risk ratio, mean difference) used in the synthesis or presentation of results.	Statistical analysis (page 3-4, line 145-150)
Synthesis methods	13a	Describe the processes used to decide which studies were eligible for each synthesis (e.g. tabulating the study intervention characteristics and comparing against the planned groups for each synthesis).	Statistical analysis (page 3-4, line 144-157)
	13b	Describe any methods required to prepare the data for presentation or synthesis, such as handling of missing summary statistics, or data conversions.	Statistical analysis (page 3-4, line 144-157)
	13c	Describe any methods used to tabulate or visually display results of individual studies and syntheses.	Statistical analysis (page 3-4, line 144-157)
	13d	Describe any methods used to synthesize results and provide a rationale for the choice(s). If meta-analysis was performed, describe the model(s), method(s) to identify the presence and extent of statistical heterogeneity, and software package(s) used.	Statistical analysis (page 3-4, line 144-157)
	13e	Describe any methods used to explore possible causes of heterogeneity among study results (e.g. subgroup analysis, meta-regression).	Statistical analysis (page 3-4, line 144-157)
	13f	Describe any sensitivity analyses conducted to assess robustness of the synthesized results.	NA
Reporting bias assessment	14	Describe any methods used to assess risk of bias due to missing results in a synthesis (arising from reporting biases).	Data extraction and quality assessment (page 3)
Certainty assessment	15	Describe any methods used to assess certainty (or confidence) in the body of evidence for an outcome.	Statistical analysis (page 3-4, line 144-157)
**RESULTS**			
Study selection	16a	Describe the results of the search and selection process, from the number of records identified in the search to the number of studies included in the review, ideally using a flow diagram.	Study characteristic in the result (page 4, line 160-170), Figure 1 PRISMA flow
	16b	Cite studies that might appear to meet the inclusion criteria, but which were excluded, and explain why they were excluded.	Study characteristic in the result (page 4, line 160-170), Figure 1 PRISMA flow
Study characteristics	17	Cite each included study and present its characteristics.	Table 1
Risk of bias in studies	18	Present assessments of risk of bias for each included study.	Table S2 and Figure S1 in supplement
Results of individual studies	19	For all outcomes, present, for each study: (a) summary statistics for each group (where appropriate) and (b) an effect estimate and its precision (e.g. confidence/credible interval), ideally using structured tables or plots.	Result (page 4-12, line 159-283), Figure 2-5, and Figure S2 in supplement
Results of syntheses	20a	For each synthesis, briefly summarise the characteristics and risk of bias among contributing studies.	Result (page 4-12, line 159-283), Figure 2-5, and Figure S2 in supplement

Section and Topic	Item #	Checklist item	Location where item is reported
	20b	Present results of all statistical syntheses conducted. If meta-analysis was done, present for each the summary estimate and its precision (e.g. confidence/credible interval) and measures of statistical heterogeneity. If comparing groups, describe the direction of the effect.	Result (page 4-12, line 159-283), Figure 2-5, and Figure S2 in supplement
	20c	Present results of all investigations of possible causes of heterogeneity among study results.	Result (page 4-12, line 159-283), Figure 2-5, and Figure S2 in supplement
	20d	Present results of all sensitivity analyses conducted to assess the robustness of the synthesized results.	NA
Reporting biases	21	Present assessments of risk of bias due to missing results (arising from reporting biases) for each synthesis assessed.	Evaluation of publication bias (page 12-13, line 284-290)
Certainty of evidence	22	Present assessments of certainty (or confidence) in the body of evidence for each outcome assessed.	Result (page 4-12, line 159-283), Figure 2-5, and Figure S2 in supplement
**DISCUSSION**			
Discussion	23a	Provide a general interpretation of the results in the context of other evidence.	Discussion (page 13-15, line 291-420)
	23b	Discuss any limitations of the evidence included in the review.	Discussion (page 15, line 395-411)
	23c	Discuss any limitations of the review processes used.	Discussion (page 15, line 395-411)
	23d	Discuss implications of the results for practice, policy, and future research.	Discussion (page 15, line 395-411)
**OTHER INFORMATION**			
Registration and protocol	24a	Provide registration information for the review, including register name and registration number, or state that the review was not registered.	Search strategy and study eligibility (page 2, line 95-96)
	24b	Indicate where the review protocol can be accessed, or state that a protocol was not prepared.	Search strategy and study eligibility (page 2, line 95-96)
	24c	Describe and explain any amendments to information provided at registration or in the protocol.	Search strategy and study eligibility (page 2, line 95-96)
Support	25	Describe sources of financial or non-financial support for the review, and the role of the funders or sponsors in the review.	Funding (page 15, line 438)
Competing interests	26	Declare any competing interests of review authors.	Conflict of interest statement for all authors (page 16, line 445)
Availability of data, code and other materials	27	Report which of the following are publicly available and where they can be found: template data collection forms; data extracted from included studies; data used for all analyses; analytic code; any other materials used in the review.	Data available statement (page 15, line 442-443)

From: Page MJ, McKenzie JE, Bossuyt PM, Boutron I, Hoffmann TC, Mulrow CD, et al. The PRISMA 2020 statement: an updated guideline for reporting systematic reviews. BMJ 2021;372:n71. doi: 10.1136/bmj.n71

Supplemental Material

**Table S1**	Search terms
**Table S2**	Summary of Risk of Bias in Non-randomized Studies – of Interventions (ROBIN-I) for included studies
**Figure S1**	Risk of bias summary for included RCTs
**Figure S2**	The change of weight from baseline

Table S1. Search terms

1	GLP-1 receptor agonist*.mp.
2	GLP-1 RA.mp.
3	Glucogon-like peptide-1 receptor agonist*.mp.
4	liraglutide.mp.
5	semaglutide.mp.
6	dulaglutide.mp.
7	lixisenatide.mp.
8	exenatide.mp.
9	albiglutide.mp.
10	efpeglenatide.mp.
11	1 or 2 or 3 or 4 or 5 or 6 or 7 or 8 or 9 or 10
12	chronic kidney disease.mp.
13	chronic renal disease.mp.
14	chronic kidney failure.mp.
15	chronic renal failure.mp.
16	chronic kidney insufficiency.mp.
17	chronic renal insufficiency.mp.
18	CKD.mp.
19	diabetic kidney disease.mp.
20	diabetic nephropathy.mp.
21	DKD.mp.
22	DN.mp.
23	12 or 13 or 14 or 15 or 16 or 17 or 18 or 19 or 20 or 21 or 22
24	advanced.mp.
25	stage 5.mp.
26	non-dialysis.mp.
27	pre-dialysis.mp.

28	glomerular filtration rate.mp.
29	GFR.mp.
30	eGFR.mp.
31	<15.mp.
32	less than 15.mp.
33	28 or 29 or 30
34	31 or 32
35	33 and 34
36	24 or 25 or 26 or 27 or 35
37	end stage kidney disease.mp.
38	ESRD.mp.
39	ESKD.mp.
40	end stage renal disease.mp.
41	hemodialysis.mp.
42	haemodialysis.mp.
43	peritoneal dialysis.mp.
44	37 or 38 or 39 or 40 or 41 or 42 or 43
45	23 and 36
46	44 or 45
47	11 and 46
48	limit 47 to humans

Table S2. Summary of Risk of Bias in Non-randomized Studies – of Interventions (ROBIN-I) for included cohort and non-randomized controlled studies

**Bias due to**	**Terawaki et al. (2013)**	**Hiramatsu et al. (2015)**	**Kondo et al. (2017)**	**Yajima et al. [1] (2018)**	**Yajima et al. [2] (2018)**	**Hirose et al. (2018)**	**Chen et al. (2022)**
Confounding	Moderate	Low	Low	Low	Low	Moderate	Moderate
Selection	Low	Low	Moderate	Low	Low	Low	Low
Classification of interventions	Low	Low	Low	Low	Low	Low	Low
Deviations from intended interventions	Low	Low	Low	Low	Low	Moderate	Moderate
Missing data	Low	Moderate	Low	Low	Low	Moderate	Moderate
Measurement outcome	Low	Low	Low	Low	Low	Low	Low
Selection of reported result	Low	Low	Low	Low	Low	Low	Low
**Overall**	**Moderate**	**Moderate**	**Moderate**	**Low**	**Low**	**Moderate**	**Moderate**

Figure S1. Risk of bias summary Cochrane risk-of-bias for randomized trials (RoB 2) for included RCTs

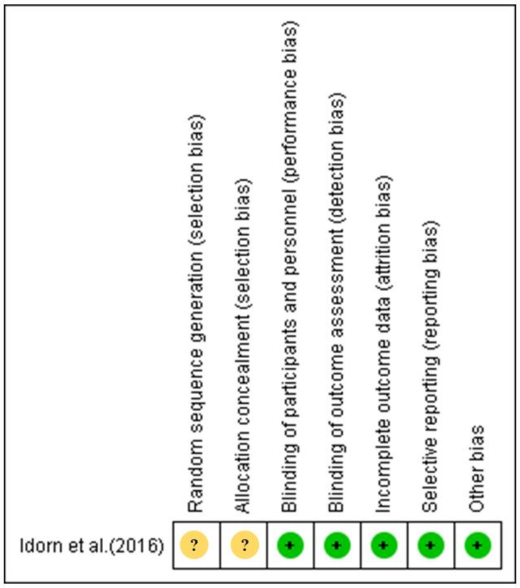

Figure S2. The change of weight from baseline

Study name	Difference in means	Standard error	Variance	Lower limit	Upper limit	Z-Value	p-Value	Relative weight
Hiramatsu et al (2015)	-3.800	6.133	37.615	-15.821	8.221	-0.620	0.536	0.37
Idorn et al (2016)	-2.200	0.440	0.194	-3.063	-1.337	-4.995	0.000	71.81
Yajima et al [1] (2018)	-2.200	0.708	0.501	-3.587	-0.813	-3.109	0.002	27.82
	-2.206	0.373	0.139	-2.937	-1.474	-5.910	0.000	

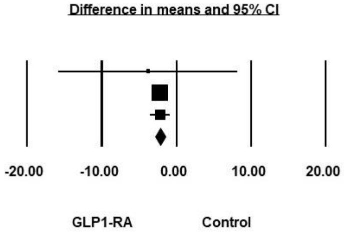

$I^2 = 0\%$
